# Evidence for Over-Dispersion in the Distribution of Clinical Malaria Episodes in Children

**DOI:** 10.1371/journal.pone.0002196

**Published:** 2008-05-21

**Authors:** Tabitha Wanja Mwangi, Gregory Fegan, Thomas Neil Williams, Sam Muchina Kinyanjui, Robert William Snow, Kevin Marsh

**Affiliations:** 1 Kenya Medical Research Institute, Centre for Geographic Medicine Research, Coast/Wellcome Trust Collaborative Program, Kilifi, Kenya; 2 Infectious Diseases Epidemiology Unit, Department of Population Health, London School of Hygiene and Tropical Medicine, London, United Kingdom; 3 Centre of Tropical Medicine, Nuffield Department of Clinical Medicine, John Radcliffe Hospital, Oxford, United Kingdom; 4 Kenya Medical Research Institute/Wellcome Trust Collaborative Program, Nairobi, Kenya; Queensland Institute of Medical Research, Australia

## Abstract

**Background:**

It may be assumed that patterns of clinical malaria in children of similar age under the same level of exposure would follow a Poisson distribution with no over-dispersion. Longitudinal studies that have been conducted over many years suggest that some children may experience more episodes of clinical malaria than would be expected. The aim of this study was to identify this group of children and investigate possible causes for this increased susceptibility.

**Methodology and Principal Findings:**

Using Poisson regression, we chose a group of children whom we designated as ‘more susceptible’ to malaria from 373 children under 10 years of age who were followed up for between 3 to 5 years from 1998–2003. About 21% of the children were categorized as ‘more susceptible’ and although they contributed only 23% of the person-time of follow-up, they experienced 55% of total clinical malaria episodes. Children that were parasite negative at all cross-sectional survey were less likely to belong to this group [AOR = 0.09, (95% CI: 0.14–0.61), p = 0.001].

**Conclusions and Significance:**

The pattern of clinical malaria episodes follows a negative binomial distribution. Use of lack of a clinical malaria episode in a certain time period as endpoints for intervention or immunological studies may not adequately distinguish groups who are more or less immune. It may be useful in such studies, in addition to the usual endpoint of the time to first episode, to include end points which take into account the total number of clinical episodes experienced per child.

## Introduction

The standard immune-epidemiological approach to studying immunity in malaria uses the lack of an episode(s) of clinical malaria over a certain period of time as a marker of ‘being protected’ [1]. It is however difficult to determine the validity of such an approach if the distribution of risk of having an episode is unknown. It might be assumed that the pattern of clinical malaria disease among children of similar age and apparent exposure history living in endemic areas would follow a Poisson distribution (no over-dispersion). This assumption is supported by results that demonstrate a progressive and homogenous decrease in malaria attacks with increasing age in children [2]–[5].

However, in Senegal, children followed up for five years were found to experience between 0–40 episodes of clinical malaria [6]. There was an unexplained high susceptibility to clinical malaria for a proportion of children with some children suffering a malaria attack every 4 to 6 weeks over many years [7], however the authors were unable to identify factors associated with this increased susceptibility [8].

Over distribution is common for many infectious organisms, for instance Woolhouse and colleagues [9] have demonstrated that 20% of the population were responsible for 80% of the transmission potential for leishmania, schistosomiasis, malaria and bacterial sexually transmitted diseases while Smith and colleagues [10] have demonstrated strong evidence of heterogeneity in malaria infections with 20% of the population carrying 80% of all infections. Although many studies note the presence of a group of children with more clinical malaria disease than others [11]–[13], this phenomenon has not been studied in any detail.

We examined a group of children that have been under malaria surveillance for between 3 to 5 years to investigate the pattern of susceptibility to clinical malaria over time and to identify factors associated with this increased susceptibility in an area of low-moderate malaria transmission in Kilifi District, Kenya.

## Results

### Evidence for over dispersion in the distribution of clinical malaria episodes

**Figure 1:**
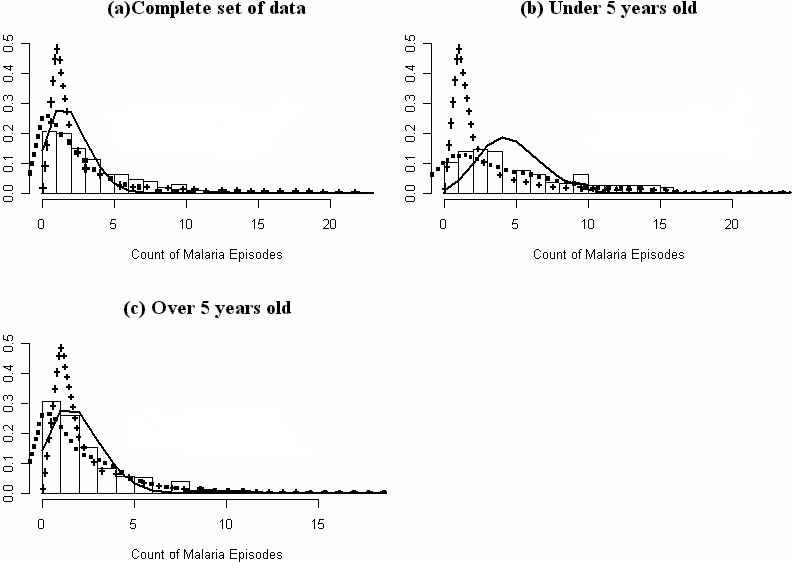
Comparing fit for the Poisson, Pareto and Negative Binomial distributions using observed total clinical malaria episodes per child as outcome. The X-axis is the total clinical episodes of malaria experienced per child and Y-axis is the proportion of children with given total disease episodes. The bars are the observed total number of cases per child, the black line is the predicted totals from the Poisson regression model, the dashed line represent the predicted total episodes from the negative binomial regression model while the crossed lines represents the predicted total episodes from the Pareto distribution. Figure (a) represents all the children, (b) all children under 5 years at the time the study started (who were followed up from 1998 to 2003) and (c) children ≥5 years of age (followed up from 1998 to 2001).

Using total malaria episodes experienced per child while controlling for mean age and time at risk, it was found that the negative binomial regression fitted better than the Poisson regression (Likelihood ratio Chi-squared test = 229.12, p<0.001). Figure 1 illustrates how the Poisson regression, negative binomial models and Pareto distribution fit the observed data and it is clear that in the two age groups, the negative binomial model fitted the observed data best. This analysis was done for different time periods and different ages (yearly graphs with age as: under 2, 2–4 and ≥5) and found to hold true (data not shown). The standard deviation(SD) of the observed data was more than double the standard deviation that would be expected if the data followed a Poisson distribution (Observed SD for children <5 years at the start of the study was 4.33 compared to the expected 1.34 whereas for children ≥5 years the observed SD was 2.41 compared to the expected 0.41).

The dispersion parameter ‘k’ was 1.9 (95% CI: 2.4–1.5). When k→∞, then the data follows the Poisson distribution. The values obtained for ‘k’ suggest moderate over-dispersion of clinical malaria episodes suggesting that there are some children who are at increased risk of clinical malaria compared to others.

**Figure 2:**
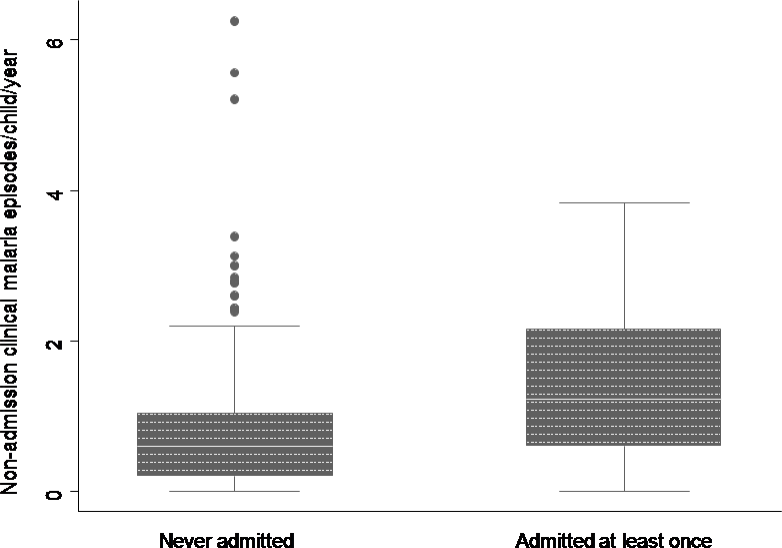
Non-admission clinical malaria episodes/child/year among children that were admitted at least once or never admitted during 3–5 years of follow-up. Box plot of the median(central line) 25%, 75% quartile ranges around the median (box width) and the upper and lower limits (T).

Children experiencing more episodes of clinical malaria appeared to be at greater risk of developing disease severe enough to require admission to hospital. Figure 2 shows that those children that were admitted had more episodes of clinical malaria per year [median: 1.4 (IQR: 0.8–2.4)] than those children that were not admitted [median 0.6 (IQR: 0.2–1.04)] and this difference was statistically significant (p<0.001). Only 51 children (13.7%) were admitted at least once in the course of this surveillance (six children were admitted twice, six admitted three times, two admitted four times and one admitted five times).

Poisson modelling was used to estimate the predicted number of clinical malaria episodes per child after controlling for mean age and time at risk. Children who experienced >2 episodes of clinical malaria above the total predicted were arbitrarily considered ‘more susceptible’. A total of 78 (21%) children comprised this ‘more susceptible’ group. Out of a total of 1,173 malaria episodes experienced during the period of the study, 55.3% was experienced by this ‘more susceptible’ group, representing only 23% of the cohort person years of follow-up. Table 1 demonstrates that these children were consistently at increased risk of clinical malaria over several years and that this phenomenon was not a result of one or two seasonal outbreaks. There was however no evidence of a difference in the incidence of non-malarial fevers in the two groups (Table 2).

**Table 1:** Episodes per child/year showing continuity of being ‘more susceptible’ over time.

Age*	Group	Year 1	Year 2	Year 3	Year 4	Year 5
Under 2	Normal	0.8	0.7	0.8	0.3	0.6
	M. susceptible	2.3	2.7	4.4	1.0	1.6
2–4 yrs	Normal	0.9	0.4	0.8	0.2	0.5
	M. susceptible	2.8	2.3	3.2	0.6	1.9
≥5 yrs (ALL)	Normal	0.6	0.4	0.4	End of follow-up	
	M. susceptible	2.6	1.6	2.2	for these children	

Note:

* Age in years at the start of the study

Year 1 = September 1998 to September 1999

Year 2 = October 1999 to October 2000

Year 3 = October 2000 to November 2001

Year 4 = November 2001 to November 2002

Year 5 = December 2002 to September 2003.

M. susceptible = ‘more susceptible’. These are the children who experienced >2 episodes of clinical malaria above the total predicted from the Poisson regression model.

**Table 2:** Factors associated with children being ‘more susceptible’ than others.

Factor	Normal	‘More Susceptible’		Crude Odds ratio	
	N = 295 (79.1%)	N = 78 (20.9%)		(95% confidence intervals)	
Female	143 (48.5%)	31 (40%)		1.42 (0.86–2.37), p = 0.2	
Bednets (N = 342)Ω	140 (52.6%)	40 (52.6%)		1.0 (0.6–1.7), p = 1.0	
Transmission*	105 (35.6%)	38 (48.7%)		1.71 (1.03–2.85), p = 0.03	
Parasitological cross-sectional survey (n = 238).					
>5,000 par/µl of blood§	13 (7.3%)	13 (22%)			3.6 (1.5–8.48), p = 0.002
Always –veβ	70 (39.1%)	7 (11.9%)			0.21 (0.08–0.5), p<0.001
Genetic markers					
Sickle trait (N = 352)	36 (13%)	4 (5.4%)			0.38 (0.13–1.12), p = 0.07
Thalassaemia (N = 284)φ	148 (67.9%)	47 (71.2%)			1.17 (0.6–2.14), p = 0.6
Incidence of clinical disease [Episodes/child/year and 95% confidence intervals]					
	Normal		‘More Susceptible’		
Malaria fevers	0.56 (0.51–0.61)		2.35 (2.17–2.53)		
Non-malarial fevers	0.91 (0.85–0.97)		0.93 (0.81–1.04)		

Note:

Ω Bednets either untreated or treated that were in good condition.

*Transmission: This reflects the household level of transmission and shows the proportion of children in the two groups that came from homes with above average parasite rate (≥50%) compared to those below average (<50%).

§ These are geometric mean parasite densities in those cross-sectional surveys were the slide was positive. The cut-off for high geometric mean parasite density was set arbitrarily at >5,000 parasites/µl of blood compared to those with less

β Compares those who were always negative at all six cross-sectional surveys with those who were positive at least once.

φ α Thalassaemia genotype: Homozygous (-α/-α) and heterozygous (αα/-α) compared to normal (αα/αα). Comparing homozygous and heterozygous alone did not make a difference to these associations.

The children in the ‘more susceptible’ group were also 4.9 times more likely to be admitted to hospital for malaria than others [Crude or Unadjusted Odds Ratio (UOR) = 4.9 (95% CI: 2.6–9.3), p<0.001).

### Risk factors associated with the ‘more susceptible’ group of children

Univariate analysis (Crude odds ratio) was used to look at associations between each individual variable and susceptibility (Table 2). We looked at the relationship between the presence and density of asymptomatic parasitaemia at all cross-sectional surveys and the chance of developing clinical episodes (the geometric mean densities were calculated from all cross-sectional surveys with a positive slide). Participants who never had a positive slide during any of the six cross-sectional bleeds were less likely to be in the ‘more susceptible’ group than those that had at least one positive slide in any of the cross-sectional surveys [UOR = 0.21 (95% CI: 0.08–0.5), p<0.001].

Among those that were parasite positive in the parasitological surveys, children that had high geometric mean parasite density (GMPD) (>5,000 parasites/µl of blood) were more likely to be ‘more susceptible’ to malaria [UOR = 4.2 (95% CI: 1.8–9.7), p<0.001]. The incidence of clinical malaria (episodes/child/year) among those slide negative at all cross-sectional surveys [0.69 (95% CI: 0.6–0.79)] was lower than that of those who had a GMPD at the cross-sectional surveys of <5,000 parasites/µl of blood [1.19 (95% CI: 1.1–1.3)] which was lower than that of those with a GMPD ≥5,000 parasites/µl of blood [1.78 (95% CI: 1.52–2.03)].

Sickle cell trait was associated with decreased risk of being in the ‘more susceptible’ group but this was not statistically significant [UOR = 0.38 (95% CI: 0.13–1.12), p = 0.07] while children from households with average parasite rate ≥50% (which indicated high exposure to malaria) were more likely to be in the ‘more susceptible’ group [UOR = 1.71 (95% CI: 1.03–2.85), p = 0.03].

Using Poisson regression and controlling for sickle trait, high geometric mean parasite density, household level of transmission as well as the clustering effect of household, there was evidence that children who were parasite negative in all six cross-sectional surveys were less likely to belong in the ‘more susceptible’ group [Adjusted Odds ratio (AOR) = 0.29 (95% CI: 0.14–0.61), p = 0.001)]. None of the other factors (including household level of transmission) showed statistically significant associations in this multiple regression.

**Figure 3:**
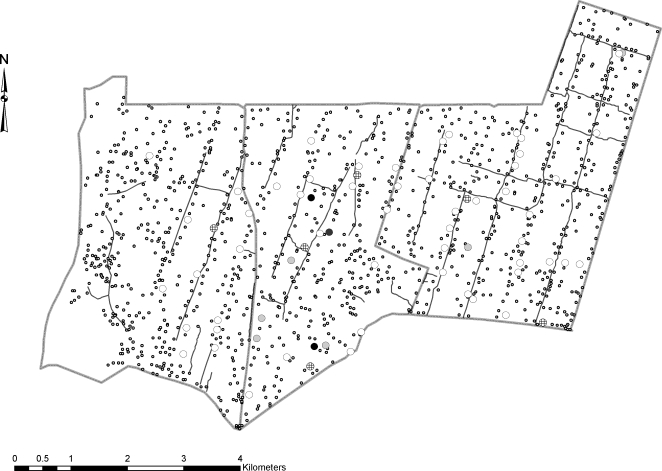
Distribution of the total number of ‘more susceptible’ children within study households. The clear circles represents the households that did not have any children ‘more susceptible’ than others, the circles with squares represent the households that had between 1–3 children ‘more susceptible’, the grey circles represent those that had 4–6 and the black circles represent those that had more than 7 children in the households ‘more susceptible’ than others. The smaller dots spread across the map are all the other households within the larger study area that were not included in the surveillance.

Using the random effects Poisson regression likelihood ratio test, there was evidence, of clustering of episodes of clinical malaria within households (p = 0.03). However, the number of households was small (n = 60) with large differences in the numbers of children per house (6.2±4.1). Half of the households did not have a single ‘more susceptible’ child but none of the households were made up purely of ‘more susceptible’ children (figure 3).

Data collected in one malaria season (4 months from May to August of 1999) was analysed to find out whether it was possible to identify this high risk group over a short period of time. Within this 4-month period, 78% of the children in the ‘more susceptible’ group experienced at least one episode of clinical malaria while only 29% of the others experienced an attack. Five children (4 from the ‘more susceptible’ group) had 3 episodes of malaria within the four months of surveillance.

**Figure 4:**
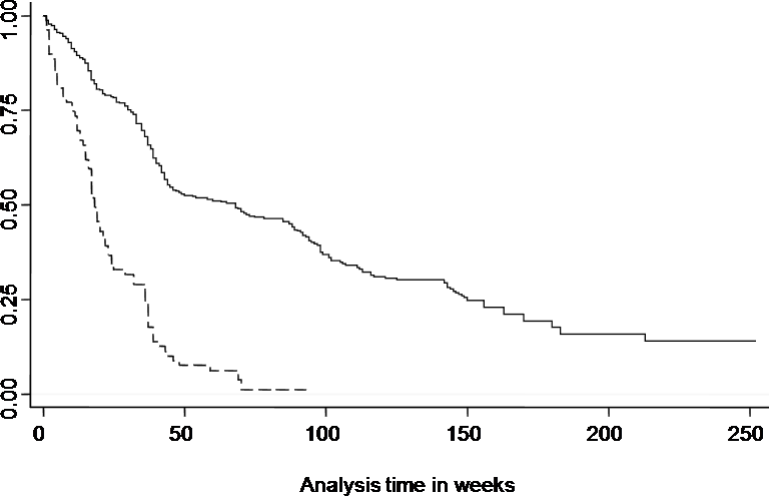
Kaplan-Meier survival curve of the time to first episode of clinical malaria. Dashed line represents the ‘more susceptible’ children and the solid line represents the time to first episode for the other children.

Figure 4 demonstrates that the ‘more susceptible’ group experienced their first clinical episode earlier and this was the same for all children irrespective of age.

### Children with no episodes of clinical malaria

A total of 77 (21%) children did not experience a single episode of clinical malaria over several years of follow-up. In this group, 23% had sickle cell trait, 49% slept under an intact bednet, 59% were over five years of age and 36% lived in houses with transmission above average (compared to 9%, 53%, 36% and 39% respectively in the others who experienced any number of clinical malaria attacks). Figure one illustrates that there were more children observed to have no clinical malaria episodes than would be expected if the data were to fit a Poisson distribution especially among those under 5 years of age.

## Discussion

We found that within a small geographical area and over several years, the risk of experiencing clinical episodes of malaria was markedly heterogenous, some children being ‘more susceptible’ than others of the same age group. This increased susceptibility appears to be malaria specific as it was not observed for non-malaria fevers.

The pattern of clinical malaria attacks followed a negative binomial distribution. For the purposes of further analysis, we arbitrarily chose children who over the study period experienced a total of more than two episodes of clinical malaria above what was expected from the Poisson distribution. This group designated as ‘more susceptible’ were also at higher risk of clinical malaria severe enough to warrant admission to hospital and some were admitted more than once. This is consistent with earlier work by Snow and colleagues [14], [15] which showed that there was a space and time clustering of severe malaria admissions and that there were children who were admitted several times with severe malaria.

We also noted that there were a large number of children who do not get any malaria episodes in the whole period of follow-up. This is a phenomenon that was also observed from 11 years of malaria surveillance in an area in the Sudan with low malaria transmission [16]. In fact in Sudan, 25% of the children under a year old at the start of that study, did not experience any episodes of malaria which was far higher than expected if episodes followed the Poisson distribution.

Children who were parasite negative at all cross-sectional surveys were less likely to belong to the ‘more susceptible’ group of children. Whilst this may seem obvious, it is important to recognise that being repeatedly negative may stem from different circumstances. Some children may be less exposed (some homes did not have a single ‘more susceptible’ child), in which case they may in fact have increased susceptibility that is not manifest due to lack of exposure to infected bites. However, the effect of local variations in exposure (measured by household parasite rates) was weak in the multiple regression model though there was evidence of spatial clustering of clinical malaria attacks. Alternatively, some children could be parasite negative in the face of continuous exposure, in which case they have genuine reduced susceptibility. Such protection could stem from a range of factors including behavioural differences between households and genetic factors which either act directly to control the risk of clinical disease or by influencing immune responses. Sickle cell trait probably provides the greatest degree of protection of any single genetic polymorphism. In this study, the odds ratio for association with the less susceptible group was consistent with this, though not statistically significant. However, it should be noted that we have previously estimated in the same group of children that sickle cell trait contributes only 2.1% of the variance in incidence of non-severe clinical malaria [17].

Children with low parasite densities at cross sectional survey (i.e. lower geometric mean densities over time) had a lower incidence of clinical malaria compared to those with higher densities, suggesting that ‘resting’ levels of asymptomatic parasitaemia may reflect the immune status of the individual. Although the “strain specific” aspects of immunity to malaria are often emphasised, such an observation would argue for a significant degree of cross protective immunity. For instance, in a study in Senegal on ten children who experienced more episodes than expected over a short time period, it was found that each new clinical episode was caused by a distinct genotype [18]. However, the fact that, as in the current study, this small group formed part of a larger group of children not experiencing multiple episodes, may indicate that in most children growth of newly inoculated parasites, regardless of “strain”, is restricted, while in a few this is not the case, leading to disease manifestation.

Whatever the underlying mechanisms, the phenomenon of increased susceptibility was evident over even short periods of time: within a 4-month period in a single malaria season, 78% of the ‘more susceptible’ group (as defined by their experience over the entire period of study) of children experienced at least one episode of clinical malaria compared with 29% in the rest of the population. However, depending on the relative size of the two groups, it may be difficult to tell these groups of children apart over a short time period. The significance of this for longitudinal studies may vary depending on the reasons for the heterogeneity observed and the questions being asked. Whatever the case, it is important to be aware that simply detecting a clinical episode of malaria in a time limited longitudinal study may not identify groups with homogenous levels of ‘protection’ and ‘susceptibility’.

It has previously been suggested that concentrating control resources on the ‘more susceptible’ children may lead to more successful control interventions [10], [19]. Whilst our findings support this idea, it remains to be established whether this could lead to practical interventions. One possibility is that under a situation where resources are limited, intermittent preventative treatment could be targeted to children presenting with malaria at health facilities, rather than at the whole childhood population in the chosen age range. In situations where clinical data can be linked with location data it may be possible to map areas with a high prevalence of clinical disease, as has been done for severe malaria admissions in some hospitals in Africa [15], [20].

In conclusion, we have confirmed over-dispersion in the distribution of clinical malaria episodes in a relatively small area. Although this may have possible implications for targeting interventions, we suggest that such heterogeneity is especially relevant to studies of both naturally acquired immunity and vaccine trials. It may be useful in such studies, in addition to the usual endpoint of the time to first episode, to include end points which take into account the total number of clinical episodes experienced per child.

## Materials and Methods

### Study participants and longitudinal surveys

The study was conducted as part of a longitudinal study defining and describing non-severe malaria among people living in Ngerenya, an area of low-moderate malaria transmission in Kilifi District on the coast of Kenya. Consent was sought from parents of 373 children <10 years of age in August-September 1998. Verbal consent was sought from village leaders and heads of households. The study was then explained in the local language to the mother or child's direct care giver. Information sheets were left with the family for discussion and the study explained again the following day and any questions answered. The mother or child's direct care giver was then asked to sign or thumb-print on a consent form to show willingness for her child/children to participate in the study after fully understanding it. They were also made aware that they could withdraw their child/children from the study at any point.

Details of the weekly surveillance, and parasitological surveys are described in Mwangi *et al*. [3]. Briefly, weekly axillary temperature measurements were taken from all study participants. Blood smears for parasitaemia measurements were taken from those with a history of fever or fever (axillary temperature ≥37.5°C). All symptomatic children with parasitaemia were treated with Sulphadoxine/ pyrimethamine which was the recommended drug for malaria treatment during the period of the study. Both active and passive case detection was used with study participants being encouraged to attend the study clinic anytime they had a fever. Data used for this analysis were collected from September 1998 to September 2003. Children who were over eight years of age in September 2001 were dropped from the study, therefore some children were followed up for a maximum of three years while others were followed up for five years.

The primary measure for analysis was episodes of clinical malaria among study participants during follow-up. Clinical malaria was defined as fever and any level of parasitaemia for children under one year old and fever accompanied by parasitaemia ≥2,500 parasites/µl of blood for children 1–10 years old [3]. If malaria episodes occurred within 21 days of each other, only the first episode was counted (episodes occurring within the next 3 weeks were considered drug failures). Sixty-nine episodes of clinical malaria were censored using this criterion.

Six parasitological surveys were conducted, half in the high transmission, wet season (June 1999, July 2000, June 2001) and half in the dry season when transmission was low (March 2000, October 2000, March 2001). Blood samples were taken for genetic and immunology studies. A study to investigate bednet condition and use was conducted in June 2000, details of which are described elsewhere [21].

Haemoglobin types (HbA, HbS) were characterized by electrophoresis using cellulose acetate gels (Helena, USA), while participants were typed for the common African 3.7-kb α-globin deletion by polymerise chain reaction [22]. In this population, HbAS was 50% protective against non-severe malaria episodes and that protection varied with age [22], [23] while α-thalassemia had no protective effect on non-severe malaria [24].

### Admissions

Admission data were collected at the paediatric wards at the Kilifi District Hospital (KDH) which is the main referral hospital for residence of the study area and would capture most admissions. All paediatric admissions to the KDH were assessed by a clinician and a standard set of laboratory parameters collected routinely [25]. Thick and thin blood films were stained with Giemsa and counted for asexual stages of *P. falciparum*. A primary diagnosis of malaria was made if the child had a positive blood film for *P. falciparum* at admission with no other detectable cause for the clinical presentation.

### Data processing and statistical methods

All records were double entered into a database (FoxPro® version 2.5) and both entries cross-checked for errors before cleaning. All data analyses were done using STATA® software, version 9.0. (Stata Corporation, Texas, USA). We generated a map using ArcGIS® version 9.0 plotting all households within the study and indicated the total number of “more susceptible” children within each study household.

Kruskal-wallis test was used to investigate the difference in the median total number of malaria episodes experienced per child among those admitted and those not admitted with malaria. Poisson regression models, negative binomial regression models and Pareto distribution were compared for fit using total episodes of clinical malaria per child for the period of follow-up as the outcome measure. These distributions and their plots were generated using the R statistical package, version 2.62 (R Foundation for Statistical Computing, Vienna, Austria. ISBN 3-900051-07-0, URL http://www.R-project.org). A better fit using the negative binomial regression model would indicate the presence of over-dispersion. We calculated a parameter that inversely measures the extent of over-dispersion, the ‘k’ factor, which is calculated using 1/α [26]. An aggregated population will have small values of ‘k’ and very highly over-dispersed data will have ‘k’<1, while Poisson distribution is obtained if k→∞.

A Poisson model was used to estimate the predicted total episodes of clinical malaria per child after controlling for mean age and time at risk. Children who experienced a total of >2 episodes of clinical malaria above what was predicted for the time at risk were considered within this analysis as ‘more susceptible’. After identifying the ‘more susceptible’ group, we sought to investigate factors related to this increased susceptibility. Crude odds ratios were used to identify these factors. Factors with a p-value <0.1 were put in a multiple Poisson regression model in order to estimate the same associations after controlling for possible confounders.

In order to investigate the presence of clustering of ‘more susceptible’ children within the households, we used the random effects Poisson regression model.

Kaplan-Meier survival curves were plotted using the time in weeks to the first episode of clinical malaria in comparing the ‘more susceptible’ group to the others.

### Determining household level transmission rates

To determine the level of malaria transmission in an individual house, parasite prevalence data from a single cross-sectional survey conducted in August-September of 1998 were used. The overall mean parasite rate among those 1–9 years of age was 43.5% (95% CI: 38–49.1%). Due to the small number of children per house in some of the homes, houses were merged into 13 zones (<2 km apart) and the parasite rate calculated. Households were then classified as above average (parasite rate ≥50%) and those average or below (parasite rate <50%).
